# The degree of hepatic arterial blood supply of portal vein tumor thrombus in patients with hepatocellular carcinoma and its impact on overall survival after transarterial chemoembolization

**DOI:** 10.18632/oncotarget.19767

**Published:** 2017-08-01

**Authors:** Juxian Sun, Jie Shi, Bin Huang, Fantian Cheng, Weixing Guo, Wan Yee Lau, Shuqun Cheng

**Affiliations:** ^1^ Department of Hepatic Surgery VI, Eastern Hepatobiliary Surgery Hospital, Second Military Medical University, Shanghai, China; ^2^ Department of Imaging, Eastern Hepatobiliary Surgery Hospital, Second Military Medical University, Shanghai, China; ^3^ Department of Hepatopancreatobiliary Surgery I, The Central Hospital of Wuhan, Tongji Medical University of Science and Technology, Wuhan, China; ^4^ Faculty of Medicine, The Chinese University of Hong Kong, Shatin, New Territories, Hong Kong SAR, China

**Keywords:** blood supply, portal vein tumor thrombus, hepatocellular carcinoma, TACE

## Abstract

**Purpose:**

To investigate the degree of arterial blood supply of portal vein tumor thrombus (PVTT) in patients with hepatocellular carcinoma (HCC), and to evaluate its impact on overall survival after transarterial chemoembolization using lipiodol +/– gelatin sponge particles (TACE).

**Results:**

Of the 10 patients who underwent surgery, the number of patients with good/mild/poor staining of PVTT by methylene blue were 3, 4, and 3, respectively. The degrees of methylene blue staining in these patients correlated well with the degrees of accumulation of lipiodol in PVTT in these patients, i.e. good/mild/poor in 3, 4, and 3 patients, respectively. For the 77 patients who underwent TACE as treatment, they were divided into 2 groups: good accumulation of lipiodol (*n* = 27) and mild/poor accumulation of lipiodol (*n* = 50) on CT. The overall median survival between the 2 groups was 10.0 months vs 2.7 months, (*p* < 0.001). Multi-variable analysis showed degree accumulation of Lipiodol (OR, 2.057; 95% CI,1.414–2.993; *p* < 0.001) to be an independent prognostic factor.

**Patients and Methods:**

Patients with HCC with PVTT who underwent surgical resection received preoperative TACE. At operation, arterial injection of methylene blue into the common hepatic artery was carried out. During the study period, other patients with unresectable HCC with PVTT were treated with TACE.

**Conclusion:**

In about 1/3 of patients with HCC with PVTT, the arterial blood supply from the hepatic artery to the PVTT was good. These patients responded better to TACE than those patients with mild/poor arterial supply.

## INTRODUCTION

Hepatocellular carcinoma (HCC) is the sixth most common cancer and the third most common cause of cancer-related death worldwide [1]. Though imaging techniques have improved, HCC is commonly diagnosed at intermediate or advanced stages. Portal vein tumor thrombus (PVTT), which is the leading cause of poor prognosis of HCC, is present in 44.0% to 62.2% of patients [2]. Controversies exist in the management of patients with HCC with PVTT [3, 4]. Sorafenib is the only drug which is recommended by EASL and AASLD for patients with PVTT bu it can only improve survival for about 3 months [5]. Resectional surgery can offer a chance of a cure, but the tumor recurrence rate is high. Transarterial chemoembolization (TACE) is a useful palliative treatment for patients with unresectable HCC and it is based on HCCs being mainly supplied by the hepatic artery. Major PVTT in patients with HCC is an important risk factor of poor prognosis, but it is no longer considered to be an absolute contraindication to TACE. In selected patients with PVTT with good hepatic reserve and good collateral circulation around the porta hepatis, TACE can safely be carried out. The 30-day mortality is reported to be lower than 1.2% since 2010 [6]. Clinically, it is more important to control the progress of PVTT than the liver tumor because as PVTT grows the main portal vein can become blocked. Complete/partial blockage of the main portal vein results in decrease blood flow to the liver with rapid deterioration in liver function, development of severed ascites, portal hypertension with bleeding esophageal haemorrhage, and rapid intra and extrahepatic metastases. For patients with HCC with PVTT, although there was no report on complete response (CR) to TACE, partial response (PR) has been reported in 19.5% to 26.3% and stationary disease (SD) 42.5% to 62.7% [7]. The median survival between responders (10.5 mo) and non-responders (5.5 mo) to TACE can differ significantly [8]. Previous study [9] has proved that there would be relationship between response of PVTT and survival after radioembolization. The aim of this study to find whether the degree of hepatic arterial blood supply of PVTT is related to overall survival after TACE for patients with HCC with PVTT.

## RESULTS

**Figure 1:**
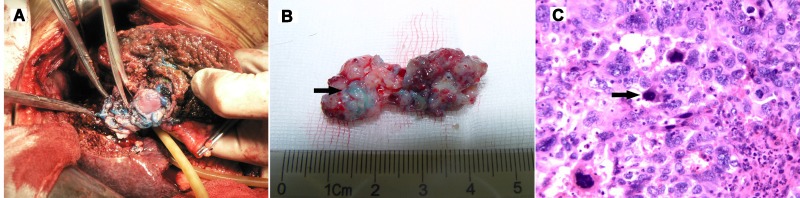
**(A)** Surgical procedure for removing PVTT. **(B)** More than 2/3 of The PVTT was dyed with methylene blue (Arrow). **(C)** Methylene blue was detected in microvessels microscopically (Arrow)

The arterial methylene blue dyeing test was carried out on 10 patients who underwent preoperative TACE 4 weeks prior to surgical resection (Figure 1A). The average length of PVTT was 2.7 cm. Of the 10 patients, the PVTT was dyed by methylene blue to be good in 3 patients, mild in 4 and poor in 3. The degrees of methylene blue staining in these patients correlated well with the degrees of accumulation of lipiodol in PVTT as shown on CT, i.e. good/mild/poor in 3, 4, and 3 patients, respectively (Table 1). Thus, more than two thirds of patients with PVTT were dyed with methylene blue (Figure 1B). Under microscopy, methylene blue was seen in small arterioles (Figure 1C). However, only about one third of the patients had good staining of PVTT by methylene blue to suggest good blood supply of PVTT coming from hepatic artery.

**Table 1:** Clinico-pathological features of the 10 patients who underwent liver resection

Number of patients	Sex (F/M)	Year	HbsAg	Tumor number	Tumor Diameter	PVTT Type	Surgical procedure	PVTT Length	Dying results (Good/Mild/Poor)
1	M	45	+	1	5 cm	III	Thrombectomy	3.2 cm	G
2	M	44	+	1	9 cm	II	En bloc	2.2 cm	P
3	M	44	+	1	3.5 cm	II	En bloc	2.7 cm	M
4	M	45	+	1	6 cm	II	En bloc	2.6 cm	M
5	M	59	+	1	6 cm	II	En bloc	1.6 cm	G
6	M	52	+	1	8 cm	II	En bloc	2.4 cm	P
7	M	40	+	1	7 cm	III	Thrombectomy	3.5 cm	M
8	M	65	+	2	6 cm	III	Thrombectomy	2.8 cm	M
9	M	50	+	2	8 cm	III	Thrombectomy	3.6 cm	P
10	M	48	+	3	8 cm	II	En bloc	2.3 cm	G

77 patients with unresectable HCC with PVTT underwent TACE for palliation. There were 69 men and 8 women with a median age of 49.8 years (range 29–73 years). 47 patients (61.0%) received one session of TACE, and 30 (39.0%) had more than one session. The median survival was 5.9 month for all the patients. When these patients were divided into 2 groups according to the degree of accumulation of Lipiodol in the PVTT as shown on CT (type IV + type III, the low accumulation group, *n* = 28 + 22 = 50; and type II + type I, the high accumulation group, *n* = 8 + 19 = 27), there were no significant differences between the 2 groups in age, gender, hepatitis serology, liver function parameters, AFP level, number of TACE sessions, tumor characteristics and PVTT classification (Table 2).

**Table 2:** Clinico-pathological Features of the 2 subgroups who underwent TACE

Variable	Lipiodol accumulation		
	Low (*n* = 50)	High (*n* = 27)	*P* Value
Age(years)	48.9 ± 9.4	51.4 ± 9.6	0.270
Gender(n),Male:Female	45/5	24/3	0.879
AFP(μg/L), < 20: ≥ 20	8/42	3/24	0.440
CEA(μg/L)	4.4 ± 13.8	3.3 ± 4.1	0.705
AST(IU/L)	76.0 ± 40.1	85.5 ± 47.3	0.362
ALT(IU/L)	62.1 ± 44.3	59.1 ± 30.3	0.754
Total bilirubin(μmol/L)	19.4 ± 9.2	19.4 ± 9.4	0.931
HBsAg(n),Positive:Negative	44/6	23/4	0.703
Tumor number(n), Single:Multiple	30/20	14/13	0.604
Tumor diameter(cm)	9.1 ± 3.3	9.0 ± 3.5	0.931
TACE sessions, 1: > 1	25/25	22/5	0.014
Classification of PVTT (n)			0.171
Type I	9	11	
Type II	21	10	
Type III	14	6	
Type IV	6	0	

**Figure 2:**
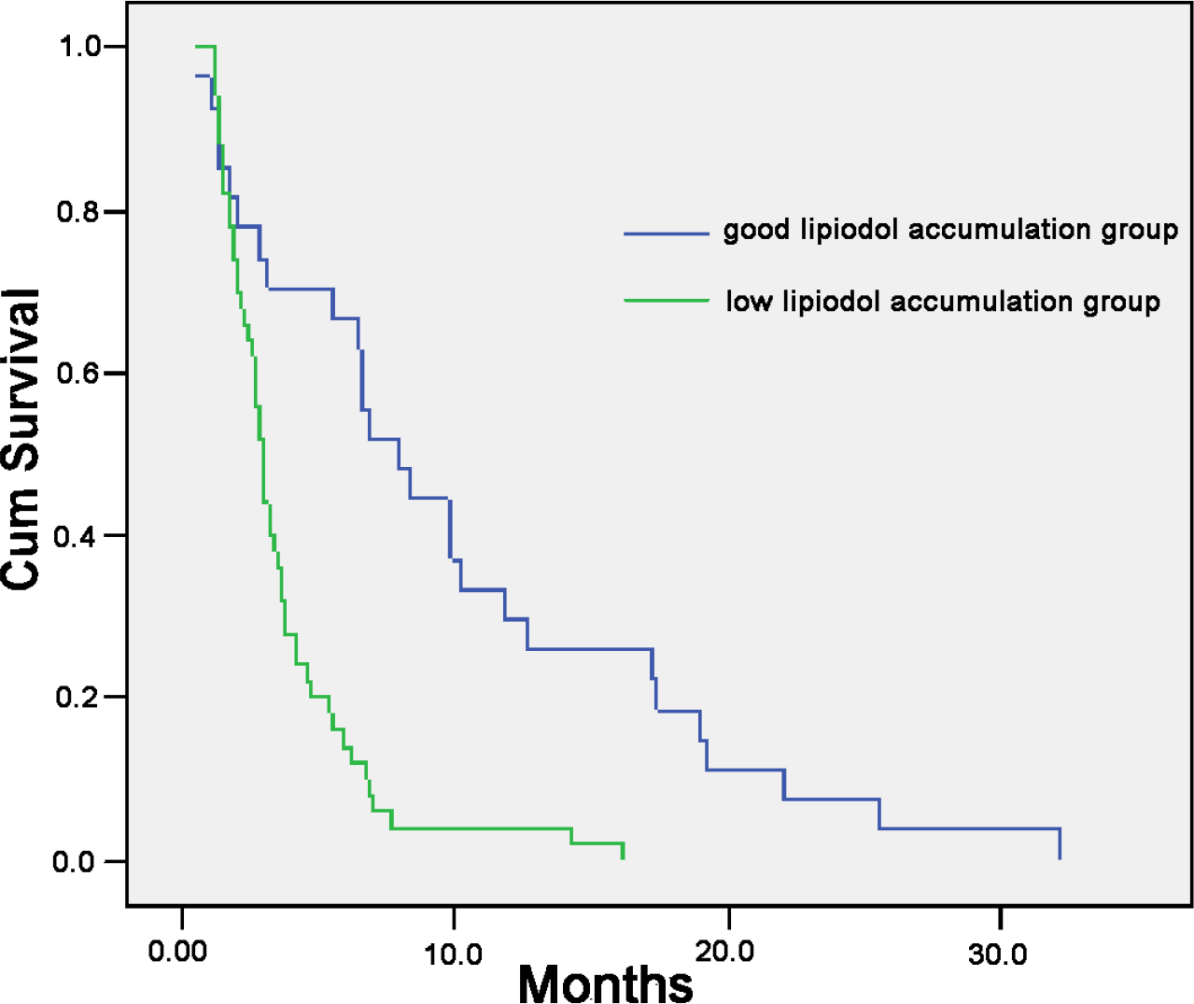
Overall survival according to different degrees of accumulation of Lipiodol in the PVTT

**Figure 3:**
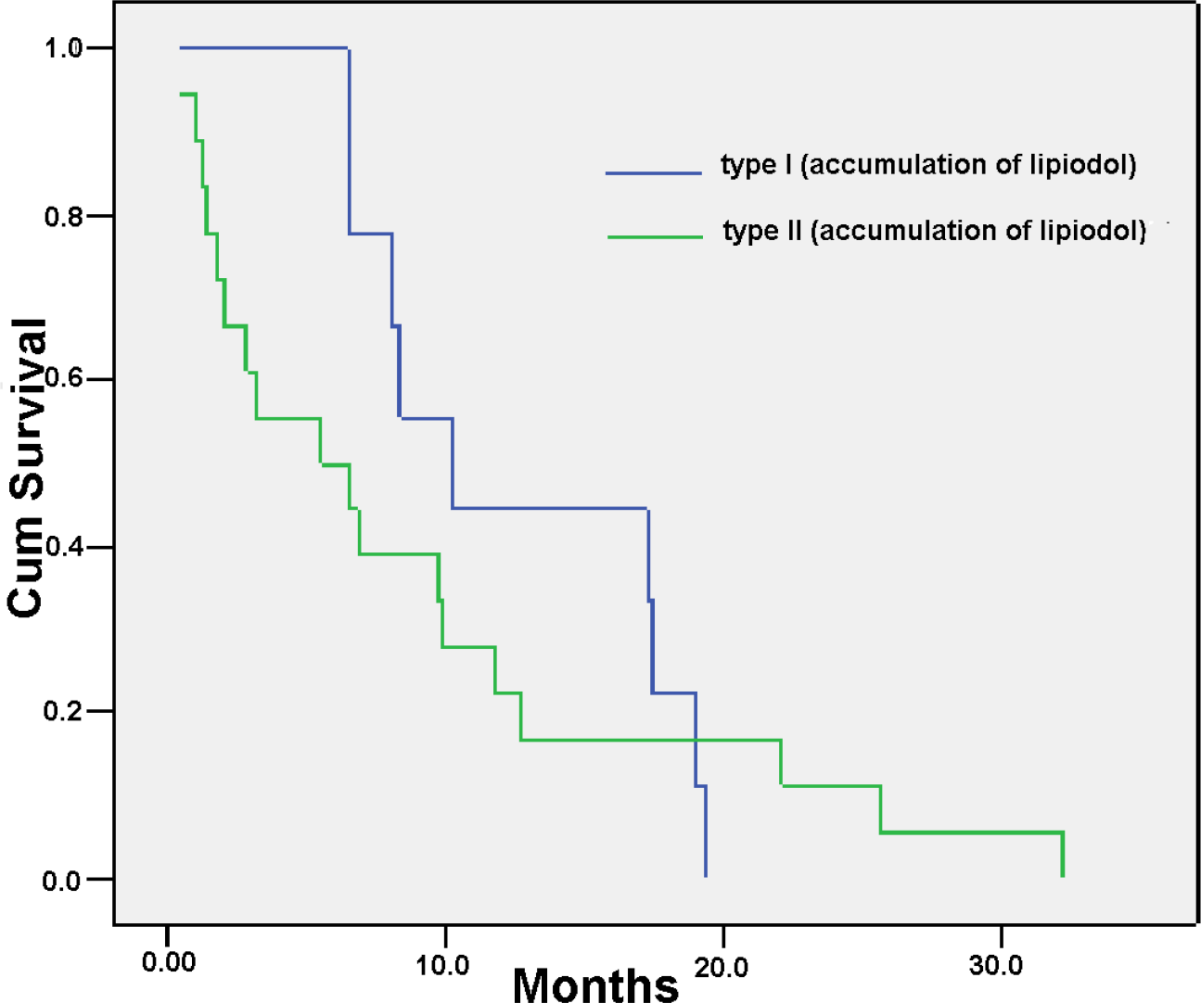
Overall survival according to different degrees of accumulation of Lipiodol in the PVTT

**Figure 4:**
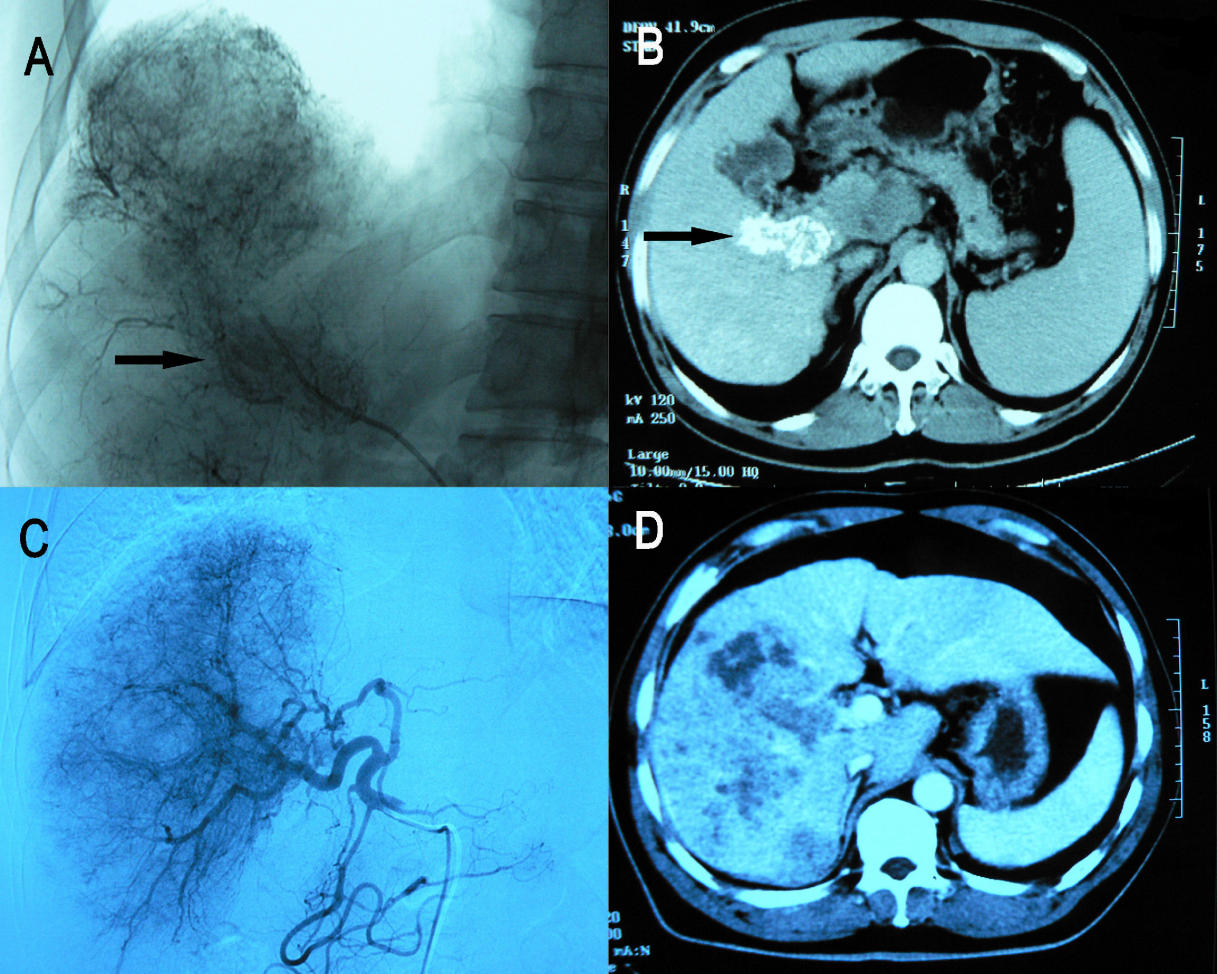
**(A** and **B)** PVTT was seen during digital subtraction angiography (DSA) and Lipiodol heavily accumulated in PVTT (OS, 17.4months); **(C** and **D)** PVTT was not seen during DSA and Lipiodol lightly accumulated in PVTT (OS, 1.5 month)

The Kaplan-Meier survival curves comparing the 77 patients with PVTT showing good accumulation of Lipiodol versus mild/poor accumulation of Lipiodol as shown on CT scan are shown in Figure 2. The overall survival of patients with types III or IV PVTT (the low lipiodol accumulation group) was significantly worse than patients with types I or II (the good lipiodol accumulation group). The median survival was 3.7 month vs. 10.0 month, respectively, *P* < 0.001). About one third of patients with PVTT. i.e. the I+II patients (*n* = 27) had significantly better overall survival than the type III + IV patients (*n* = 50) after TACE. There is also a tendency that the overall survival of type I was longer than type II patients (median survival 12.5 month vs. 8.7 month, HR 2.252, *P* = 0.089) (Figure 3). Thus, TACE produced the best survival in patients with PVTT which showed homogeneous accumulation of lipiodol on CT scan (Figure 4).

Survival of the 4 classes of lipiodol classification according to the 4 classes of PVTT are shown in Table 3.

**Table 3:** Survival of the 4 classes of lipiodol classification according to the 4 types of PVTT

PVTT extent class	Lipiodol classification	N. patients	Median Survival (Month)
Tpye I	1	2	13.5
	2	9	11.0
	3	6	5.8
	4	3	3.2
Tpye II	1	4	13.4
	2	6	6.4
	3	11	3.6
	4	10	2.7
Tpye III	1	3	10.7
	2	3	6.6
	3	3	2.4
	4	11	4.8
Tpye IV	1	0	NA
	2	0	NA
	3	2	3.3
	4	4	2.2

Univariable analysis was used to evaluate any possible correlations between survival and 11 dichotomized variables for all the 77 patients (Table 4). Results show that both tumor diameter and degree of accumulation of Lipiodol are independent prognostic factors. In multivariate analysis, tumor diameter (HR, 1.106; 95% CI,1.011–2.211; *P* = 0.029) ,degree of accumulation of Lipiodol (HR, 2.057; 95% CI,1.414–2.993; *P* = 0.001) and AST (HR, 1.107; 95% CI,1.008–1.027; *P* = 0.002) were found to be independent prognostic factors. After making a sensitivity analysis by forcing the type of PVTT into the model, we found that the type of PVTT was not an independent prognostic factor (HR, 1.426; 95% CI,0.901–2.258; *P* = 0.130).

**Table 4:** Univariate analysis of prognostic factors

Variable	*n*		Median survival (months)	HR	95%CI	*P*	
Age, years, < 60: ≥ 60	47/30	10.2/8.9		0.996	0.969–1.024		0.119
Gender,Male:Female	69/8	6.0/5.1		0.952	0.442–2.383		0.706
AFP, μg/L, < 20: ≥ 20	12/65	6.2/5.9		1.000	1.000–1.001		0.885
ALT, IU/L, < 44: ≥ 44	26/51	5.3/6.0		0.998	0.989–1.006		0.368
AST, IU/L, < 44: ≥ 44	16/61	4.18/6.4		1.008	1.000–1.016		0.063
Albumin,g/l, < 35: ≥ 35	14/63	4.26/6.31		0.902	0.761–1.069		0.236
Total bilirubin,μmol/L, < 17.1: ≥ 17.1	38/39	5.6/6.3		1.009	0.983–1.037		0.615
HBsAg,Positive : Negative	67/10	5.9/4.3		0.829	0.376–1.827		0.423
Tumor number, Single:Multiple	43/34	5.7/6.1		0.817	0.491–1.359		0.804
Diameter(cm), ≤ 5: > 5	11/66	10.2/5.4		1.096	1.022–1.175		0.031
TACE sessions (*n*), 1 : > 1	47/30	6.6/4.8		0.863	0.610–1.222		0.226
Classification of PVTT (*n*)				1.241	0.927–1.661		0.111
Type I	20	8.5					
Type II	31	5.1					
Type III	20	5.6					
Type IV	6	2.5					
Accumulation of Lipiodol (*n*)				1.733	1.347–2.231		0.000
I	9	12.5					
II	18	8.7					
III	22	4.0					
IV	28	3.5					

## MATERIALS AND METHODS

### Patients

From July 2010 to January 2012, all patients with HCC with PVTT who were treated in the Department of Hepatic Surgery VI at the Eastern Hepatobiliary Surgery Hospital were included into this study, retrospectively. The extent of PVTT was classified into: type I, involvement of segmental/sectoral branches of portal vein; type II, involvement of left or right portal vein; type III, involvement of main portal vein; and type IV, involvement of superior mesenteric vein.

Patients with resectable HCC with PVTT were offered surgical resection. The inclusion criteria for surgical resection were: 1) resectable HCC; 2) Type II/III PVTT; 3) Child-Pugh A liver function, 4) no hepatic vein invasion and/or extrahepatic spread. These patients were treated with preoperative TACE 4 weeks before surgery. The degree of lipiodol accumulation in PVTT was assessed as in the patients who had unresectable HCC with PVTT treated by TACE (please see latter part of the study). All patients were given detailed information on the pros and cons of surgical resection and TACE, including treatment efficacies and potential adverse effects. Written informed consent was obtained from all the patients for their data to be used for research purposes. The study was approved by the Ethics Committee of the Eastern Hepatobiliary Surgery Hospital.

### The surgical procedure

Surgery was performed through a right subcostal incision with a midline extension. Intraoperative ultrasound was done as a routine to confirm the preoperative findings. After mobilization of the liver and dissection of the structures in the porta hepatis, the proper hepatic artery was isolated and and 20 ml of methylene blue (Shanghai X-Y Biotechnology) was injected through the artery using a 23 guage needle. Pringle’s maneuver was then applied to occlude the blood inflow of the liver using continuous clamping. The planned liver resection was carried out in the usual manner. All grossly detected lesions were resected. The PVTT was surgically treated according to the location and extent of PVTT. For 6 patients with a PVTT which was located within the resected region, the PVTT was resected en bloc with the tumor. For 4 patients with a PVTT which had extended beyond the resection line, the main portal vein was opened and the PVTT was extracted from the portal vein. After flushing with normal saline and confirming that no PVTT remained, the incision in the portal vein was closed with a continuous 5–0 prolene suture.The porta hepatis clamp time in all the patients was less than 30 minutes.

The resected specimens were studied by two pathologists. For each pathologist, photographs of the transverse, sagittal and coronal sections of the specimens were taken on the liver tumor and on the PVTT. These photographs were then compared with a background showing a grid with one millimeter squares. The areas which had been dyed with methylene blue were counted from the whole PVTT areas on the 3 sections. If more than 2 sections were stained more than 50% of the areas, then the arterial blood supply of PVTT was classified as good. If only one section was stained more than 50% of the areas, then the arterial blood supply of PVTT was classified as mild. Otherwise, they were classified as poor. The average of the two values taken by the 2 pathologists was used as the final value.

### The TACE procedure

During the study period, patients with unresectable HCC with PVTT underwent TACE. The following patients were included into this study: 1) unresectable HCC with PVTT; 2) type II/III PVTT; 3) Child-Pugh liver function A or B; 4) no hepatic vein or inferior vena cava invasion; 4) no previous treatment for HCC and no other malignancies.

TACE was performed using the Seldinger technique. Celiac and superior mesenteric arteriograms were routinely performed to show the hepatic arterial and portal venous anatomy. The catheter was then inserted to the tumor-feeding branches of the hepatic artery. Selective arteriograms were performed to demonstrate the feeding artery of PVTT. In patients with an arterioportal shunt, embolization with gelatin sponge particles was performed to occlude the shunt. Mitomycin C(MMC) 20 mg, hydroxycamptothecine 10mg and Lipiodol 10–30 ml (at 1–2 ml/cm of the tumor diameter) were then given. In patients with type III PVTT, care was taken not to completely block the hepatic artery.

CT scans were taken a day following TACE. The accumulation of lipiodol in the PVTT was classified as type 1, homogeneous; type II, slight defective; type III, inhomogeneous; and type IV, only slight accumulation^10^.

### Follow up

All patients were followed-up by the same team of clinicians. Post-treatment follow-up including ultrasonography, serum biochemistry and AFP levels were done once every 1 to 2 months. Contrast CT scan and chest X-ray were carried out once every 3 months for surveillance of tumor progression or metastasis. If residual viable tumor was detected on dynamic CT imaging, TACE was repeated in individuals who had compensated liver function.

### Statistical methods

The x^2^ test or Fisher exact test (2-tailed) was used to compare categorical data, and the student’s *t* test was used for continuous data. Overall survival were calculated using the Kaplan-Meier method. Factors that appeared to be associated with survival on univariable analysis (*p* < 0.1) were introduced into the multivariable Cox proportional hazard model to determine the adjusted risk ratio. All statistical analyses were carried out using SPSS version 13.0 for Windows (SPSS Inc.; Chicago, IL). For all the tests, a *P* value < 0.05 was considered statistically significant.

## DISCUSSION

Controversies exist in the management of patients with HCC with PVTT. Sorafenib is the only drug that improves survival in patients with advanced HCC for about 3 months. However, a SHARP trial demonstrated that tumor response to sorafenib treatment is only 2%–3% [11]. So consensus in China proposes comprehensive treatment of PVTT including TACE because evidence indicates that TACE can be performed safely and feasibly in select PVTT patients. The median OS obtained with sorafenib was 8.1 months in patients with PVTT in the SHARP, and the median OS was only 5.9 months in our study. We found that there are significant differences between different PVTT types, the median OS PVTT Type I patients was 8.5months and the Type IV was only 2.5 months. So we consider that there might be differences in PVTT types and tumor characters between these two studies.

The origin where the blood supply of PVTT comes from is unclear and possible sources include the hepatic artery, portal vein or other vessels around the porta hepatis. This study showed that in about one third of patients with HCC, the blood supply to PVTT comes mainly from the hepatic artery. In these patients, TACE is more efficacious in prolonging survival than those patients who had poor blood supply to PVTT from the hepatic artery. And in the surgical part, we validated that the flow of PVTT was in line with the lipiodol accumulation.

TACE is widely used in advanced hepatocellular carcinoma. In selected patients with good hepatic reserve and collateral circulation around the porta hepatis, it can be used in patients with HCC with PVTT. Georgiades [12] et al demonstrated that TACE was safe and effective for patients with HCC that had invaded the main portal vein. The 1-month mortality was 0% and the median survival was 9.5months. There was no TACE-related hepatic infarction or liver failure. Jia and his associates [13] showed that the degree of accumulation of Lipiodol in PVTT after TACE was an independent prognostic factor. Our study supported that a higher degree of Lipiodol accumulation in PVTT was associated with significantly better overall survival after TACE.

Evidence is now accumulating that the blood supply of PVTT is complicated. Imaging techniques like enhanced CT or ultrasound suggest that at least some blood supply comes from the hepatic artery. The exact percentage of the blood supply is unknown and it seems to vary amongst individuals. Our study showed that in about one third of patients with HCC with PVTT the blood supply comes mainly from the hepatic artery. For the other two thirds of patients, the blood supply probably comes mainly from the portal vein or from other vessels in the porta hepatis. Published data suggested that the tumor itself can form new vessels which come from differentiation of cancer stem cells [14]. Our study probably explains why only about one third of patients with PVTT responded well to TACE.

Many factors are related to Lipiodol accumulation including [15, 16, 17] blood supply, structures of capillaries inside the tumor and clearance rate of Lipiodol by tumor cell et al. Generally, blood supply is considered to be one of the most important factors contributed to early Lipiodol accumulation. Also it was reported^15^ that patients with good tumor vascularity are generally considered to have good lipiodol accumulation and therefore, a good prognosis. Our method of carrying out TACE is to use gelatin sponge particles and lipiodol as embolizing agents. Gelatin sponge particles produce temporary occlusion of tumor feeding vessels which lasts for about two weeks [18, 19]. Lipiodol, when injected into the hepatic artery, selectively remains more in tumor nodules for several weeks to over a year due to siphoning effect from hypervascularization of tumor vessels and absence of Kupffer cells inside tumor tissues [20, 21]. This results in the embolic effects on small vessels. However, lipiodol, when injected into the hepatic artery of normal liver parenchyma, accumulates in the portal vessels by arterioportal communications and is gradually released into the systemic circulation via the hepatic sinusoids or undergoes phagocytosis by Kupffer cells. Thus, our study presently partly means that responding patients do better than non-responding patients which are mainly related to blood flow of the PVTT. The lipiodol is usually cleared within a week [20, 22, 23]. Thus, the way that we carried out preoperative TACE in the 10 patients with resectable HCC with PVTT should not affect the subsequent study using intrahepatic arterial injection of methylene blue to study the arterial blood supply of PVTT in the resected specimens.

This paper has limitations. Firstly, we only consider only from the aspect of blood supply in the study and we will do further research in the future ;secondly, the sample size is small and in the majority of our patients, the HCC was hepatitis B related; lastly, the accuracy in measuring the dyed area is not very high. . Whether our results can be used in HCC due to other etiologies is unknown.

## CONCLUSIONS

In conclusion, in about one third of patients, the blood supply of PVTT came from the hepatic artery. These patients responded better to TACE.

## ETHICAL APPROVAL

Not needed.
